# Emerging Parameters Justifying a Revised Quality Concept for Cow Milk

**DOI:** 10.3390/foods13111650

**Published:** 2024-05-25

**Authors:** Matteo Mezzetti, Matilde Maria Passamonti, Margherita Dall’Asta, Giuseppe Bertoni, Erminio Trevisi, Paolo Ajmone Marsan

**Affiliations:** 1 Dipartimento di Scienze Animali, della Nutrizione e degli Alimenti (DIANA), Facoltà di Scienze Agrarie, Alimentari e Ambientali, Università Cattolica del Sacro Cuore, 29122 Piacenza, Italy; matteo.mezzetti@unicatt.it (M.M.); matildemaria.passamonti1@unicatt.it (M.M.P.); margherita.dallasta@unicatt.it (M.D.); giuseppe.bertoni@unicatt.it (G.B.); erminio.trevisi@unicatt.it (E.T.); 2 Romeo and Enrica Invernizzi Research Center for Sustainable Dairy Production of the Università Cattolica del Sacro Cuore (CREI), 29122 Piacenza, Italy

**Keywords:** cheesemaking, composition, environment, flavor, human health, sustainability

## Abstract

Milk has become a staple food product globally. Traditionally, milk quality assessment has been primarily focused on hygiene and composition to ensure its safety for consumption and processing. However, in recent years, the concept of milk quality has expanded to encompass a broader range of factors. Consumers now also consider animal welfare, environmental impact, and the presence of additional beneficial components in milk when assessing its quality. This shifting consumer demand has led to increased attention on the overall production and sourcing practices of milk. Reflecting on this trend, this review critically explores such novel quality parameters, offering insights into how such practices meet the modern consumer’s holistic expectations. The multifaceted aspects of milk quality are examined, revealing the intertwined relationship between milk safety, compositional integrity, and the additional health benefits provided by milk’s bioactive properties. By embracing sustainable farming practices, dairy farmers and processors are encouraged not only to fulfill but to anticipate consumer standards for premium milk quality. This comprehensive approach to milk quality underscores the necessity of adapting dairy production to address the evolving nutritional landscape and consumption patterns.

## 1. Introduction

Milk is a liquid mixture of water, fat, proteins, carbohydrates, minerals, and vitamins that is secreted by the mammary gland of mammals to feed their offspring [1]. Milk stands out as an exceptionally nutrient-rich food, one of evolution’s solutions to ensuring the survival and fitness of thousands of animal species. It is vital for human infants and remains beneficial throughout adulthood. Evidence of this is found in the human genome, where a strong signal of positive selection is observed on the mutation that maintains lactase activity into adult life [2,3,4,5]. This evolutionary signature, which is closely linked to the domestication of livestock and the practice of milking, underscores the adaptive advantage of being able to digest milk throughout one’s lifetime. After domestication, humans selected dairy animals and developed rearing systems aimed at improving desired functional traits. Cows’ ability to produce a large amount of milk and to adapt to different environments allowed them to have the greatest success among dairy species [6,7], to the point of becoming the main producers of milk that is consumed fresh or transformed into dairy products worldwide [8]. For this reason, the term “milk” is only used to refer to cow milk, while the animal from which milk originates must always be stated for other dairy species.

Nowadays, cow milk and derived products have become staple food products in most modern societies. Milk quality is mainly assessed at the farm level by the stakeholders along the milk production chain. The “entry level” for any dairy farm is providing a product that is hygienically safe and free from any undesired compounds with detrimental effects on human health or negatively affecting the technological properties of milk (i.e., poisonous compounds, antibiotics, harmful bacteria, water).

In several countries, the payment to the milk supplier farms is adjusted by a system of bonuses/penalties based on a panel of milk compositional traits that deeply impact the milk properties (i.e., cheese yield, coagulation features, and flavor). Besides these macronutrients, milk contains minerals, vitamins, and several other minor compounds that are recognized to affect human health and technological processes but are currently not considered by any “traditional” quality-based payment system. Furthermore, the consumer’s concern for animal welfare and environmental impact has grown considerably over the past few decades. Thus, the modern consumer’s perception of milk quality has expanded beyond the traditional safety and compositional traits. Several novel milk quality characteristics encompass a highly dynamical system of management, feeding, and genetic strategies, implemented along the production process. Any effort made by milk suppliers to meet the novel requirements is often related to increased milk production costs at the farm level and should thus be adequately supported by payment systems along the supply chain. This review explores the rationale behind revising the concept of milk quality. A literature review was conducted on the main quality aspects that are attributed to cow milk, considering both research and review articles published in peer-reviewed journals. Quality aspects were considered for inclusion only when their implementation depended on management practices adopted at the farm level. Any milk quality aspect attributed to technological treatments adopted between the milk collection at the farm and final consumption was not covered by the present review. The final aim is to encourage milk supply chain stakeholders in developing quality-based payments rewarding the supplier farms that fulfil the quality requirements of modern consumers.

## 2. Milk Safety: An Entry-Level Requirement of the Milk Quality Concept

Providing a product that is safe and healthy for the final consumer and that does not contain undesired substances that affect the technological processes occurring along the milk production chain is the primary requirement for any dairy farm. Detrimental effects for human health have been reported for several substances acting as potential milk pollutants. Pollutants can be grouped into biotic and abiotic factors. The first class includes living organisms developing during the milk production process or storage phase, currently monitored by the dairy industry, and reflected by the milk’s total bacterial count (TBC). A heightened TBC in milk makes a relevant contribution in affecting milk’s shelf life, transformation process, and lastly, the health of the final consumer (especially for some harmful bacterial strains). Factors impacting on the TBC at the bulk tank are primarily related to hygienical practices adopted between milking and the collection of the milk. Despite that, raw milk is not considered for direct human consumption in most countries in the world, and several sanitation practices are currently adopted to lower the risks related to milk contamination by living bacteria. Thus, biotic pollutants revest mainly a food science relevance, and they will not be considered further in the present review. Conversely, the load of abiotic pollutants in milk is the result of previous contaminations hindering the mammary gland, the environment, or the feeds offered to the cow. Several abiotic pollutants have a tremendous damaging power on human health, and monitoring and containing the risk of milk contamination by these substances is the remit of international authorities for food safety (Table 1). Among these, the heavy metal levels in liquid milk and dairy products represent a relevant risk factor for human health in several parts of the world [9]. Several phenomena concur in determining milk contamination with heavy metals, including the contamination of soils where the feedstuffs offered to cows grow [10]. Most heavy metals (i.e., lead, cadmium, mercury, nickel, aluminum) are non-essential elements causing toxic effects even at very low concentrations [11,12,13]. For lead and cadmium in particular, adverse effects have been documented in pregnant women due to their ability to cross the placental barrier and affect fetal development [14,15]. Other harmful milk pollutants include dioxins, which exert carcinogenic effects [16] and represent a relevant risk factor in milk due to their ability to bioaccumulate through the food chain [17,18].

Monitoring the contamination of milk by other classes of pollutants is required of dairy industries, as these substances have a milder effect on human health or mainly exert a negative effect on milk’s quality characteristics. Bacterial toxins include a heterogeneous class of compounds released by several bacterial strains, mainly after intramammary infections, but also after contamination of the feedstuffs offered to the cow. Despite detrimental effects on human health being attributed to these compounds [19,20,21,22], no safety threshold currently exists for the concentration of any specific bacterial toxin in milk. Instead, the risk of contamination of milk for most of these compounds is indirectly assessed by the dairy industry though monitoring milk’s somatic cell count (SCC). The rationale behind this practice in cow milk is that bacterial toxins mostly arise from mastitis-causing bacteria. SCCs serve as a reliable indicator of the leukocyte activities taking place in the mammary gland [23], and a heightened SCC could thus reflect intramammary infections. Besides the negative effects attributed to bacterial toxins, even metabolites produced by leukocytes that are consequential to the immune response negatively affect milk characteristics. Among these, heat-stable proteases (plasmin) and lipases (lipoprotein lipase) released by activated leukocytes are known for detrimentally affecting the coagulation features of milk, causing casein and fat degradation during storage and producing off-flavors in liquid milk [24,25]. Furthermore, leukocyte activities taking place in the udder increase the permeability of the mammary epithelium, resulting in a heightened flow of metabolites in the bloodstream following the concentration gradient. Typically, this is reflected by increased electrolytes and a lowered lactose concentration in milk [26], and those changes could affect the cheesemaking aptitude [27]. Thus, SCC thresholds are commonly included in any quality-based payment system worldwide [28]. The contamination of milk by several other classes of pollutants does not induce any leukocyte activation in the mammary gland, forcing the dairy industry to account for other specific biomarkers besides SCCs to assess them.

Mycotoxins are produced by fungi during the life cycle of plants. These compounds accumulate in feed, negatively affecting animal health and altering several metabolic functions when included in dairy rations at high levels [29]. Most of the dietary mycotoxins ingested by ruminants represent a minor risk factor for milk quality aspects, as they are directly inactivated by rumen bacteria once ingested by the cow [30]. The main exception to this general rule is aflatoxin B1, a mycotoxin produced by *Aspergillus flavus* in several cereals [31]. In the rumen, aflatoxin B1 is converted to aflatoxin M1 by bacteria, and the latter accumulate in milk [32]. Negative effects on human health are attributed to aflatoxin metabolites, and safety thresholds for these compounds in milk are currently adopted worldwide (safety thresholds range between 0.05 and 0.5 ppb depending on the country in question) [33]. 

Other pollutants that are routinely monitored by the dairy industry are drug residuals (mainly antibiotics). These represent a risk to human health due to their potential role in selecting antibiotic-resistant bacterial strains [34]. Despite that, the main reason behind their close monitoring by the dairy industry is the detrimental effects that antibiotic residuals contained in milk could exert on bacterial strains that are essential for cheesemaking processes (mainly *Lactobacillus)* [35]. In the consumer’s perception, terms like “natural” and “antibiotic-free” are gaining attention, with organic milk often being perceived as aligning the closest with these health-related qualities [36,37]. This perception stems from stringent restrictions on the use of antibiotics in organic farming, but it is not underpinned by clearly defined bioactive mechanisms that are attributable to antibiotic residues in milk. A legal framework (Regulation EU 2019/6) governs the responsible use of veterinary medicinal products in conventional farms in Europe, and similar regulations are adopted in dairy farms located in most other countries in the world. These frameworks ensure responsible use through strict prescription and administration rules, along with mandatory withdrawal periods. These practices suggest that organic certification may not necessarily translate to additional health benefits regarding antibiotic residues in milk [38].

Another important factor considered by the dairy industry in several countries is the contamination of milk with pollutants that have a potential harmful effect on cheese maturation and aging. These include bacterial spores, primary resulting from a dietary contamination of milk by spore-forming bacteria (i.e., *Bacillus* and *Clostridium*), the last of which is introduced by silage and is responsible for the blowing defect of aged cheeses [39,40]. Altogether, these safety parameters represent the entry-level requirement for the milk quality concept, and they should always be met before considering any other milk characteristics.

**Table 1:** Main abiotic pollutants potentially found in commercial cow milk, their origin, factors affecting milk contamination, and potential implications for human health.

Pollutant	Origin	Adverse Effect On Human Health	Reference
Mycotoxins			
Aflatoxin M1	Nutrition Feeds contaminated by *Aspergillus flavus*	Carcinogenic, genotoxic, mutagenic, and teratogenic	[19,41]
Bacterial toxins			
Cereulide	Nutrition Feeds contaminated by *Bacillus cereus* (toxin is stable during cow digestion and milk thermal treatments)	Causes emesis and nausea (poisonous effects at 0.01–1.28 µg/g)	[20,42,43]
Toxic shock syndrome toxin	Intramammary infections by *Staphilococcus aureus*	Induces fever, hypotension, congestion in multiple organs, and lethal shock	[21,44]
Shiga toxin	Intramammary infections by *Escherichia coli* (risk in raw milk)	Induces hemolytic uremic syndrome	[22,45]
Heavy metals			
Lead (Pb)	Nutrition Feeds grown on contaminated soils	Can cross the placental barrier. In utero exposure can affect fetal brain differentiation, causing neurotoxic effects (decreased intelligence quotient, memory reduction, and language disturbance)	[11,12,13,14,15]
Cadmium (Cd)	Nutrition Feeds grown on contaminated soils	Negatively affects nutritional quality of milk through altering its nutritional profile; can cross the placental barrier. In utero exposure can affect fetal brain differentiation, causing neurotoxic effect (decreased intelligence quotient, memory reduction, and language disturbance). Affects reproductive systems (disturbance of androgen–estrogen balance and steroidal hormone levels). Increases breast cancer risk	[14,46,47,48]
Mercury (Hg)	Nutrition Feeds grown on contaminated soils	Negatively affects nutritional quality of milk through altering its nutritional profile	[14,48]
Pesticides			
Dioxins	Nutrition Bioaccumulates through the food chain	Human carcinogen. Causes atherosclerosis, hypertension, and diabetes	[16]
Others			
Drug residuals	Management Wrong drug suspension time	Increased risk of selecting resistant bacterial strains	[34]

## 3. Milk Quality as Reflected in Compositional Traits: Classical Principles and Novel Aspects

Compositional traits that are considered in “conventional” quality-based payment systems include the butterfat, protein, lactose, and casein concentration of milk. The reason driving this choice is the relevance of the fat, protein, and lactose concentration in defining the milk’s energy content (around 63 kcal/100 g [49]) and the pivotal role exerted by the fat and casein concentration in determining the efficiency of cheesemaking processes [50]. Cheese consists of a calcium paracaseinate reticulum entrapping fat globules and a part of the soluble phase of milk [51]. The cheese yield depends on the milk’s total fat and protein contents (i.e., fat + protein) [52] but also on the ratio between the protein and fat fractions (i.e., protein-to-fat ratio, PFR). Curds obtained from high-PFR milk contain less fat and greater percentages of protein, Ca, and P. Conversely, milk with a low PFR leads to curds containing lower levels of moisture in nonfat substances. Thus, curds obtained from low-PFR milk have a lower syneresis (serum that is lost during the pressing phase) compared to curds obtained from high-PFR milk [53]. Despite the relevance of these “classical” traits in defining milk quality being widely accepted, several other compositional traits have important implications on human health and the technological properties and flavor of milk, suggesting their contents to be promising parameters to include in a revised milk quality concept.

### 3.1. Milk Fat Composition and Fatty Acid Profile

Milk fat is of the greatest importance in determining the energy content of milk and is a vehicle for several lipophilic compounds affecting, either positively or negatively, human health (i.e., lipophilic vitamins and some pollutants) [54]. Thus, the total fat content is already included in most of the milk quality-based payment systems worldwide. In a human diet, milk fat represents a relevant dietary source for several fatty acids (FAs). Although assessing the effect of a single FA constituting milk fat is a difficult task, bioactive impacts on human health, on the shelf life of liquid milk, and on the response to maturation and aging processes of cheeses have been attributed to several FAs constituting milk fat [55,56,57]. Furthermore, several FAs have a primary role in determining the flavor (i.e., taste + aroma) of dairy products through being associated with several aromatic compounds contained in fresh milk (see following sections) or undergoing oxidation-driven alterations in liquid milk during the technological processes and in cheeses during maturation [58]. Among the saturated FAs (SFAs), the main measured FAs (C12:0, C14:0, and C16:0) potentially exert atherogenic and thrombogenic effects [59,60]. Conversely, C 18:0 does not seem to have any relevant effects on health, while other milk short-chain SFAs (i.e., C4:0 [61]) would mediate a moderately protective effect on coronary risk [55,62,63]. Thus, the effect of milk SFAs on cardiovascular risk promotion is still a topic of debate [64], as the different FA profiles of the milk fat may mediate different effects on human health [65]. Among the unsaturated FAs (UFAs), oleic acid represents the main monounsaturated FA (MUFA), while linoleic acid (LA) is the main n-6, and α-linolenic acid (ALA) is the main n-3 polyunsaturated FA (PUFAs) contained in milk in normal conditions. Enriching the UFA fraction included in the FA profile of dairy products could lower the potential harmful effects attributed to SFAs (which are reduced). Besides health implications for the consumer, lowering the SFA/UFA ratio in milk has the potential to modify cheese’s texture through lowering the melting point of the milk fat and increasing the migration of fat to the rind during the cheese-pressing phase. In cow milk, this primarily depends on the oleic/palmitic acid ratio of the milk fat, as these are the major SFAs and UFAs included in the FA profile with high and low melting points, respectively [66]. Among the UFAs, increasing milk’s PUFAs has the greatest potential for improving human health, as the latter include conjugated linoleic acid (CLA), n-6, and n-3 essential FAs. Conjugated linoleic acid is a collective term for several isomers of linoleic acid. Milk and dairy products are the main sources of CLAs in the human diet, mostly due to their high rumenic acid (*cis-9*, *trans-11 C 18:2*, representing 90% of CLA in cow milk) and *trans-10*, *cis-12 C18:2* contents [67]. Beneficial effects attributed to CLAs include improved immune system function and weight regulation [68], although the final effect on the consumer could depend on the relative abundance of each isomer constituting the CLA mixture [69]. Besides CLAs, other PUFAs with a fundamental role in human health are n-6 and n-3 PUFAs. Current recommendations suggest maintaining dietary n-6/n-3 ratios between 2.5 and 4:1 to reduce cardiovascular disease incidences, while the ratio normally ranges from 15:1 to 16.7:1 [70,71]. The average n-6/n3 ratio of milk fat is around 6:1 [55], and thus, lowering the n6/n3 ratio of milk (i.e., the LA/ALA ratio) has the potential to improve the health of consumers [72]. In fact, long-chained n-3 PUFAs, which are essential for human health (i.e., eicosapentaenoic—EPA—and docosahexaenoic acids—DHA), could be synthesized in humans from ALA elongation by ∆5 and ∆6 desaturase [73], despite this metabolic pathway having been documented to have a moderate efficiency in adult human beings [74]. Individual cow traits (i.e., stage of lactation, metabolic disease incidence) and several management practices (i.e., breeding strategies and nutrition plan) could affect the FA profile at the farm level, suggesting that it as a parameter for extending the definition of milk quality (Table 2).

**Table 2:** Average content and origin of the fatty acids included in fresh cow milk and their potential implications for the milk quality concept.

Nutrient	UM ^1^	Content	Origin ^2^	Implications ^3^	Reference
Total fat	g	3.0–3.9		(H+) Facilitates the dissolving and absorption of lipophilic vitamin; (H−) Several pollutants (i.e., cereulide, dioxins) accumulate in the milk fat fraction; (F+) Carrier for taste and aroma.	[42,54,55,75]
SFA	%TF	68.72		(H−) Excessively high share of SFAs in one’s diet (particularly C12, 14, and 16) may cause atherosclerosis and promote cardiovascular risk; (H+) SFAs other than C12, 14, and 16 neutralize the hyperlipidemic effect of these acids through increasing the HDL level;	[55,60]
				(SL+) Greatest stability against oxidation;	[55]
				(CT−) Cheeses are harder, less creamy, and elastic.	[66]
Butyric (C4:0)	%TF	2.87	DN	(H+) Protects against food allergy, inflammation, oxidative stress, and diabetes; interacts with GIT microbiota.	[55,76]
Caproic (C6:0)	%TF	2.01	DN		
Caprylic (C8:0)	%TF	1.39	DN		
Capric (C10:0)	%TF	3.03	DN		
Lauric (C12:0)	%TF	3.64	DN	(H−) Increase blood LDL. Contribute to determining AI and TI of milk.	[55,59,60,76]
Myristic (C14:0)	%TF	10.92	DN		
Palmitic (C16:0)	%TF	28.7	PF		
Stearic (C18:0)	%TF	11.23	PF		
MUFAs	%TF	27.4		(H+) Do not cause accumulation of cholesterol as SFAs do; have a positive effect on the concentration of HDL and reduce the abundance of LDL; (SL+/−) Do not turn rancid as readily as PUFAs; (CT+) Cheeses are softer, creamier, and more elastic.	[55,66]
Myristoleic (C14:1, cis 9)	%TF	1	DN		[55,76]
Palmitoleic (C16:1, cis-9)	%TF	1.5	PF		[55,76,77]
Oleic (C18:1, cis-9)	%TF	22.36	PF		
Vaccenic (C18:1, trans-11)	%TF	1.5–5	PF	(H+) Precursor of CLA in human organism.	[55,76]
PUFAs	%TF	4.05		(H+/−) Involved in eicosanoid production; (SL−) Greatest susceptibility to oxidation processes;	[55]
n-6 PUFAs	%TF	2.83		(H−) Excessive amounts are commonly included in human diets. Proinflammatory (stimulates series II prostaglandin release);	[55,72]
LA *(C18:2, cis-9, cis-12)*	%TF	2.57	PF	(H−) Compete with ALA for the same enzymatic system (δ-6-desaturase), inhibiting ALA elongation from forming EPA and DHA;	[55,76,78]
CLAs	%TF	0.57	PF	(H+) Immunostimulatory, anti-inflammatory, antihypertensive, anticarcinogenic, and antiatherogenic;	[55,69,76,79,80,81,82,83]
*C18:2, cis-9, trans-11*	%CLA	90		(H+) Increase LDL-to-HDL cholesterol ratio and total-cholesterol-to-HDL cholesterol ratio;	
*C18:2, trans-10, cis-12*	%CLA	1–10		(H+) Anti-adipogenic: protect against obesity through promoting a reduction in body weight. Decrease LDL-to-HDL cholesterol ratio and total-cholesterol-to-HDL cholesterol ratio;	
n-3 PUFAs	%TF	0.56		(H+) Low amounts are commonly included in human diets. Anti-inflammatory, antioxidant, protect the cardiovascular and nervous systems. Reduce lipid deposition. Protect against cancer, heart diseases, thrombosis, arterial hypertension, hyperlipidemia, senile dementia, Alzheimer’s disease, depression, rheumatoid arthritis, and skin diseases (i.e., psoriasis, acne, lupus erythematosus);	[55,78,84,85,86]
*ALA (C18:3, cis-9, cis-12, cis-15)*	%TF	0.5	PF	(H+) Could be used for EPA and DHA synthesis (especially in infants).	[55,76,87]

^1^ per 100 g of whole milk; TF: total fat; CLA is conjugated linoleic acid; ^2^ DN: De-Novo; PF: preformed; ^3^ H: human health; SL: milk shelf life; CT: cheese texture; F: flavor; (−) and (+) represent detrimental and beneficial effect, respectively; SFAs: saturated fatty acids; HDLs: high-density lipoproteins; GIT: gastrointestinal tract; UFAs: unsaturated fatty acids; MUFAs: monounsaturated fatty acids; PUFAs: polyunsaturated fatty acids; AI: atherogenic index; TI: thrombogenic index; ALA: α-linolenic acid; EPA: eicosapentaenoic acid; DHA: docosahexaenoic acid; LA: linoleic acid; FA: fatty acid.

### 3.2. Composition of the Protein Fractions

Total protein includes caseins and whey proteins, and together, these two fractions revest a high relevance for the nutritional value of liquid milk [54,88,89]. Milk protein provides all the amino acids (AAs) that are essential to a human diet, except for cysteine [90], even though the essential role documented for this AA is limited to particular physio-pathological conditions (i.e., in patients affected by oncological diseases or in cases of methionine deficiencies) [91]. Besides the total protein concentration, milk’s casein percentage has a primary role during the cheesemaking processes, closely reflecting the final cheese yield [51]. Thus, the total protein and casein concentrations are currently included in the “classical” milk quality-based payment systems. Conversely, the composition of the casein and whey protein fractions is scarcely considered as a milk quality indicator, despite several components of these two fractions differentially affecting human health, milk flavor, and cheesemaking process. The composition of the casein fraction deeply affects the cheese yield of milk [51]. β, κ, and αs1 fractions have the strongest positive relation in this respect [92,93]. A higher relative β-casein abundance improves curd formation, while higher relative κ and αS1-caseins abundances increase casein’s affinity with the rennet, thereby improving the recovery rate of milk components from the whey fraction to the curd [93]. Several components of the whey protein fraction exert a beneficial effect on human health, mainly consisting in immune-related (i.e., lactoferrin, lysozyme, lactoperoxidase, immunoglobulins—Igs—proteose peptones) and antioxidant functions (i.e., sulfhydryl oxidase and superoxide dismutase) [54,90,94], and they also marginally affect cheese yield. Minor effects in this respect have been attributed to proteose peptone, due to its emulsifying and foaming actions on other milk solids [95], but the greatest interest is attracted by the α-lactalbumin and β-lactoglobulin relative abundances. Cipolat-Gotet et al. (2018) reported α-lactalbumin to be positively associated and β-lactoglobulin to be negatively related to all the traits related to the cheese-making process. Besides affecting cheesemaking, β-lactoglobulin can affect milk’s flavor during preservation due to its high methionine and cysteine contents, as the metabolic transformation of these sulfur-containing AAs could provide an unsuitable flavor in milk [96]. The negative impact of β-lactoglobulin on the cheesemaking process and milk flavor could be attenuated in cows with a genetic variant for this whey protein (i.e., β-lactoglobulin B), as the variant encodes for a lowered β-lactoglobulin content of milk [92]. The relative abundances of the components of casein and whey protein fractions primarily depend on the breed raised and on the genetic selection criteria adopted in the herd, but they are also marginally affected by the stage of the lactation cycle of the cows. Thus, investigating the profile of caseins and whey protein fractions could be considered a promising marker for developing an extended milk quality concept.

Besides those well-documented effects related to the composition of protein fractions, another emerging topic involving both caseins and whey proteins is the generation of bioactive peptides during their enzymatic digestion along the human gastrointestinal tract (GIT) or due to the metabolic transformation driven by lactic acid bacteria (both in milk and in the human gut). Several peptides have positive effects on human health: they regulate mineral bioavailability by acting as carriers or chelators, have antithrombotic and antihypertensive action, exert positive effects on the nervous system (agonistic and antagonistic opioid peptides), and stimulate the immune system [90,97]. Conversely, other protein fragments are potential allergens and antigens, negatively affecting the health of susceptible consumers through causing adverse immune reactions [98]. In this respect, the most relevant example is represented by peptides originating from β-casein digestion. β-caseins exist under two genetic variants: A1 and A2. The two variants differ from each other through the presence of either histidine (His67) in A1 or proline (Pro67) in A2 at position 67 of the protein. Although the His67 within A1 is susceptible to proteolytic cleavage during digestion, the Pro67 within A2 is not. Thus, A1s have the potential to release short β-casomorphin (BCM) opioid peptides, including BCM-7, during gastrointestinal digestion [99]. Several authors associated BCM-7 with adverse immune reactions, an increased cancer risk, and impaired lactose digestion in milk consumers [100,101], although this remains a highly debated topic within the scientific community. In fact, many bioactive effects that were attributed to protein fragments were assessed in vitro and in animal models, and further robust investigations supporting those effects to be maintained in humans are required to justify any effort to move milk production processes in this direction. Furthermore, ameliorating human health through modifying the milk protein profile is a difficult task to accomplish, due to the vast genetic variability affecting milk protein fractions and the individual-specific mechanisms behind the generation of peptide fragments (Table 3).

**Table 3:** The average content and origin of the components of caseins and whey protein fractions and potential implications for the milk quality concept.

Nutrient	UM ^1^	Content	Origin	Implications ^2^	Reference
Total protein	g	3.3–5.4		(H+) Generates bioactive peptides regulating several metabolic functions; (H−) Some proteins or their metabolites are involved in milk allergies.	[6,54,90,98,102,103]
Caseins	%TP	80		(H+) Precursors of several bioactive peptides; (H−) Main allergen contained in milk;	[6,54,90,98]
α-s1	%TP	32		(H+) Generates bioactive peptides with antihypertensive, opioid-agonistic, immunomodulating, and antimicrobial actions that regulate mineral bioavailability;	[90,98,104]
α-s2	%TP	10		(H+) Generates bioactive peptides with antihypertensive, immunomodulating, and antimicrobial actions and regulate mineral bioavailability;	[90,98,104]
Β	%TP	28			
Κ	%TP	10		(H+) Generates bioactive peptides with antihypertensive, antithrombotic, opioid-agonistic, and antagonistic action.	[90,98,104,105]
Whey proteins	%TP	20		(H−) Stimulates proinflammatory cytokine secretion by cultured PBMCs;	[6,54]
α-lactalbumin	%TP	5	Synthesized in the mammary gland	(H+) Coenzyme in the biosynthesis of lactose; binds metal ions. Generates bioactive peptides with immunomodulatory, antihypertensive, and humoral effects (modulates cortisol and serotonin secretion). Anticarcinogenic; (H−) Third-most important milk allergen;	[90,98]
β-lactoglobulin	%TP	10		(H+) Binds calcium, FAs, and vitamins (A, D), facilitating their absorption; generates bioactive peptides with antihypertensive, antithrombotic, opioid, antimicrobial, immunomodulant, hypocholesterolemic, and radical scavenging properties; anticarcinogenic; (H−) Second-most important milk allergen;	[98,106]
Serum albumin	%TP	1	Comes from blood	(H+) Anticarcinogenic action; generates peptides with antihypertensive effects; (H−) Fourth-most important milk allergen; (F+) Carrier for taste and aroma;	[90,98,107]
Lactoferrin	%WP	<1		(H+) Iron-chelating glycoprotein, which plays an important role in iron absorption in the intestine; antimicrobial, anti-inflammatory, anticarcinogenic, immunomodulatory, and bone growth factor properties; (H−) Sixth-most important milk allergen;	[98,108]
Igs	%WP	9–15		(H+) Potential preventive action against infant diseases; (H−) Fifth-most important milk allergen.	[90,98,107,109]
Lysozyme	%WP	<1		(H+) Bactericidal action (breaks bacterial cell walls);	[54,94,98]
Lactoperoxidase	%WP	<1		(H+) Along with lactoferrin and lysozyme comprises the non-immunoglobulin protective system in milk;	[90,107]
Sulfhydryl oxidase	%WP	<1		(H+) Antioxidant enzyme;	[90]
Superoxide dismutase	%WP	<1			
Γ-casein	%TP	Traces	Hydrolysis of β-casein by endogenous enzymes	(H+) Generates bioactive peptides with antihypertensive, antithrombotic, opioid agonistic, and antagonistic action;	[90,98,104,105]
Proteose peptones	%WP	Traces	Hydrolysis of α-s1 and β-casein by endogenous enzymes	(H+) Lipolysis inhibition, antimicrobial;	[95,98,110]
Casein-macropeptides	%WP	<10	Hydrolysis of κ-casein by chymosin	(H+) Useful for diets aimed at controlling liver diseases and when branched chain amino acids are used as a carbon source. Generates peptides with opioid and satiety effects; responsible for absorption of calcium, iron, and zinc; (H−) Responsible for ketosis induction in infants.	[90,98,111]

^1^ per 100 g of whole milk; TP: total protein; WP: whey protein; ^2^ H: human health; SL: milk shelf life; CT: cheese texture; F: flavor; (−) and (+) represent a detrimental and beneficial effect, respectively; Ig: immunoglobulin; FA: fatty acid; PBMCs: peripheral blood mononuclear cells.

### 3.3. Lactose

Milk’s lactose concentration is currently included among the compositional traits that are assessed by the dairy industry, despite its concentration not being considered by any milk quality payment system. Lactose contributes to determining the total energy and the sweet taste of fresh milk [112]. Even though it represents an important energy source for humans, it may exert potentially harmful effects for those developing a deficit in lactase enzymatic activity [113]. Concerning this specific nutrient, no intervention implemented at the farm level will impact milk’s lactose content, as this sugar maintains quite stable concentrations in liquid milk from healthy cows (i.e., 4.4–5.6 g/100 g [54]). This is due to its osmotic role in determining the milk volume [114]. Thus, lactose intolerance in milk consumers (of which the worldwide prevalence has been estimated to be around 70% [115]) can only be managed by technological processes aimed at obtaining low-lactose or lactose-free milk. Nevertheless, stable lactose concentration contained in milk from healthy cows indicates that assessing milk’s lactose concentration at the bulk milk tank could be used as a promising quality parameter. Combined with other compositional traits, lactose could reflect adulteration practices (i.e., water addition) [116] and, together with milk’s electrolyte concentration and SCC, could serve as a promising marker for the early detection of a heightened subclinical mastitis incidence in the herd [26].

### 3.4. Minerals

To date, minerals are not included in the standard parameters that are assessed by the dairy industry, and their concentration is not considered by any quality-based payment system. Milk minerals affect human health and contribute to determining milk’s coagulation features and cheese texture (Table 4), besides determining the salty taste of fresh milk [112]. Milk and dairy products have a fundamental role in supplying human diets with Ca and P, due to the high concentration of these minerals in milk [89]. Those two minerals have a fundamental role in human bone metabolism: an adequate and balanced intake of Ca and P during skeletal growth increases the bone mineral density and represents a limiting factor for achieving optimal peak bone mass [117,118,119], while preventing bone loss and osteoporotic fractures in the elderly [120,121]. Unfortunately, no intervention implemented at the farm level can impact milk’s Ca and P contents. Milk contains several electrolytes (Na, K, and Cl), and as mentioned in previous sections, the concentration of these minerals could be used to reflect the health status of the mammary gland and improve the early detection of subclinical mastitis in the herd [122], preventing alteration of other parameters that are directly involved in affecting cheesemaking features due to mastitis onset. Furthermore, several microminerals included in milk (i.e., Zn and Se) have potential health implications on the final consumer due to the enzyme-cofactorial role that they exert in many biochemical processes [54,123]. In humans, an adequate intake of these minerals lowers the risk of cancer, cardiovascular disease, and nervous system alteration [124]. Despite milk contributing to a tiny portion of the daily recommended intake of most of these minerals for humans, the micromineral concentration is a relevant parameter to include in an extended milk quality concept. In fact, milk’s Se concentration varies markedly based on the dietary strategies adopted in the herd [88], while those of Zn and I are affected by several management practices adopted in the supplier farm. These include diets, sanitation practices used for teat dipping (mainly for I), seasonal trends, and farming systems used [54,125]. Thus, including the concentration of microminerals among milk quality factors could encourage the adoption of strategies effectively ameliorating their content in milk.

**Table 4:** The average content of the main minerals included in fresh cow milk, their daily recommended intake in a human diet, and relationship with milk quality properties.

Component	UM	Content ^1^	Requirement ^2^	Implications ^3^	Reference
Ca	mg	112–123	210–1300	(H+) Structural roles: skeletal development, maintaining the excitability of tissues, conduction of nerves and muscular cells, and normal blood pressure. Promotes blood coagulation processes. (CT+) Contributes to cheese mineralization and promotes cheese firmness.	[54,56,117,118,120,121]
P	mg	59–119	-	(H+) Essential for bone deposition. (CT+) Contributes to cheese mineralization and promotion of cheese firmness; involved in the physiochemical stability of the whey proteins.	[54,56,119]
Mg	mg	7–12	26–260	(H+) Functions as a cofactor of many enzymes involved in energy metabolism, protein synthesis, RNA and DNA synthesis, and maintenance of the electrical potential of nervous tissues and cell membranes.	[54]
Na	mg	42–58	-	(CT+) Involved in the physical–chemical stability of the whey proteins.	[54]
K	mg	106–163	-	(CT+) Contributes to the cheese’s physical–chemical properties, the microorganism’s selection, and enzymatic activities throughout maturation processes.	[54]
Cl	mg	80–90	-		
Zn	mg	0.5	1.1–20	(H+) Cofactor in many enzymes with a variety of biochemical functions in the living organism.	[54,126]
I	mg	0.031	0.09–0.2	(H+) Synthesis of thyroid hormones.	[54,125]
Se	mg	0.001–0.0017	0.006–0.042	(H+) Enzymatic cofactor that enhances immunity and protection against oxidative damage. (H−) Excessive consumption can lead to gastrointestinal disturbances, skin lesions, liver cirrhosis, and pulmonary edema.	[54,127,128,129]
Fe	mg	0.03–0.1	3.9–65.4	(H+) Cofactors in many enzymes and have a variety of biochemical functions in the living organism. (H−) Excess levels may become toxic to human health.	[11,54,123]
Cu	mg	Traces	-		

^1^ per 100 g of whole milk; ^2^ recommended daily intake as defined in (World Health Organization, 2004), for the lesser and most demanding human physiological phase, respectively; ^3^ H: human health; CT: cheese texture; (−) and (+) represent a detrimental and beneficial effect, respectively.

### 3.5. Vitamins

Milk’s vitamin content has scarcely been considered as a quality factor to date, despite these compounds having a beneficial role in human health (Table 5). Processed milk contains very low amounts of vitamins E, K, C, and D, mostly due to the degradation of these vitamins during technological processes (e.g., pasteurization) aimed at the preservation of milk [54,88,130], and their concentration cannot be increased by any intervention implemented at the farm level. Conversely, milk and dairy products included in the human diet represent a relevant source of the lipophilic vitamin A and of several of the hydrophilic B-complex vitamins [131]. Vitamin A is involved in visual function, sustains fetal development and embryonic growth [132], and promotes cellular differentiation in epithelial cells and bone tissue [133]. It also keeps the immune system active, strengthens its humoral and cellular components [134], and improves protein synthesis, metabolism, and cell proliferation, acting as an effective age delayer [135]. A lack of vitamin A leads to night blindness; xerophthalmia (progressive blindness due to drying of the cornea); keratinization of the digestive, respiratory, and urinary–genital tract tissues; and exhaustion and death [136]. B-complex vitamins are primarily synthesized by rumen bacteria [137,138], and milk obtained from ruminant species has a fundamental role as a dietary source of these vitamins in the human diet. Thiamine (B1) acts as cofactor in transketolation reactions of the pentose–phosphate pathway, and pyruvate dehydrogenase catalyzes oxidative decarboxylation, taking a fundamental part in several metabolic pathways [139]. Its deficiency can severely affect the cardiovascular, nervous, and immune systems (Beriberi or Wernicke–Korsakoff syndrome) [140,141]. Riboflavin (B2) has antioxidant effects and is a precursor for coenzymes that are involved in cell respiration, being involved in numerous metabolic pathways (i.e., fat, protein, and carbohydrate metabolism). Its deficiency may be linked to developmental abnormalities, growth delay, cardiac disease, and anemia [142]. Niacin (B3) is a cofactor for enzymes that are involved in several oxidoreductive reactions, and its deficiency causes a condition called pellagra [143]. Pyridoxine (B6) has an antioxidant role [144], is involved in cell signaling, and acts as a cofactor in several metabolic reactions [145]. Folic acid (B9) serves as a cofactor in several biochemical reactions that are critical for nucleic acid synthesis and is therefore necessary for cellular proliferation and survival. Also, this vitamin has a protective effect against neural tube defects, ischemic events, and cancer. Vitamin B9 deficiency decreases lymphocyte proliferative responses and natural killer cell activity [146,147,148]. Cobalamin (B12) acts as a coenzyme for transmethylation reactions with important functions in cell division, and its deficiency is related to pernicious anemia and neuropathy onset [149]. Several management practices adopted by farms (primarily nutrition), but also external factors, such as the season and individual rumen fermentation patterns of the cows, significantly impact the concentration of A and B-complex vitamins in milk, suggesting them as relevant aspects to consider in an extended milk quality concept. Some researchers [54] have attributed the higher vitamin content and distinctive FA profile in organic milk to the feed regimen adopted by organic farms (based predominantly on farm-produced, certified organic feed, and allowing for consistent pasture access for cows). It could be argued that these variations in milk composition are primarily due to a greater inclusion of green grass in the diet, rather than being inherent advantages of the organic system itself.

**Table 5:** The average content and origin of the vitamins contained in fresh cow milk, their daily recommended intake in the human diet, and their positive effects on human health.

Component	UM	Content ^1^	Requirement ^2^	Origin	Health Benefits	Reference
Lipophilic						
Pro-vitamins *Carotene*	µg	14		Synthesized by plants	Involved in retinol synthesis	[150]
A Vitamin *Retinol*	µg	36–41	375–850	Carotenoids	Involved in visual function, fetal development, cellular differentiation, immune system function, tissue repairing, and age delay	[54,132,133,134,135,136,151]
D Vitamins	µg	0.08	5–15		Promotes Ca and P absorption in the gut, regulates Ca and P homeostasis and bone mineralization and remodeling during growth, and affects innate and adaptive immune system functioning. Protects against cancer development and progression. An absolute minimum 25(OH)D level of 20 ng/mL is recommended. A lack of vitamin D causes osteomalacia (in adults) and rickets (in infants). Chronic lack is the cause of secondary hyperparathyroidism	[54,152,153,154,155,156,157,158,159]
*Ergocalciferol*				Synthesized by fungi and certain plants		
*Cholecalciferol*				Synthesized in the animals’ skin under the effect of the sun		
E Vitamins	µg	20–184	Controversial	Synthesized by plants	Antioxidant: protects lipids contained in cell membranes against oxidative damage driven by free radicals. A modulator of signal transduction and regulator of genetic expression in different signaling pathways (i.e., inhibition of the proliferation of smooth muscle cells, platelet adhesion, and aggregation of adhesion molecules). Essential in preventing coronary artery disease and atherosclerosis. Signs of vitamin E deficiency include ataxia, retinopathy, peripheral neuropathy, musculoskeletal myopathy, and immune response disorders	[54,160,161,162,163]
*Tocopherol*						
*Tocotrienol*						
K Vitamins	µg	1.1–3.2	5–65	Synthesized by plants and microorganisms	Maintenance of normal coagulation	[54,150]
*Phylloquinone*						
*Menaquinone*						
Hydrophilic						
B vitamins				Synthesized by plants and microorganisms	Essential for normal growth and reproduction. Implicated as cofactors in anabolic and catabolic reactions	[54,131,164]
*Thiamine (B1)*	µg	28–90	200–1500	Synthesized by plants and microorganisms	Cofactor of enzymes linked to the metabolism of carbohydrates and branched-chain amino acids. Important for the synthesis of nucleic acid precursor, myelin, and neurotransmitters	[54,131,139,140,141]
*Riboflavin (B2)*	µg	116–230	300–1600	Synthesized by plants and microorganisms	Precursor of flavin mononucleotide and flavin adenine dinucleotide. Riboflavin acts as an antioxidant for a healthy immune system	[54,131,142]
*Niacin (B3)*	µg	130–200	200–1700	Synthesized by rumen and microorganisms	Cofactor of oxidoreductase enzymes involved in glycolysis, lipid metabolism, protein metabolism, and detoxifying processes	[54,143,165]
*Pantothenic acid (B5)*	µg	580	1700–7000		Constituent of coenzyme A and phosphopantetheine involved in FA metabolism	[150]
*Pyridoxine (B6)*	µg	30–70	100–2000	Synthesized by plants and microorganisms	Antioxidant. Coenzyme of many reactions in metabolism of amino acids (i.e., decarboxylation and transamination), lipids, and glucogenesis. It is also involved in metabolizing carbohydrates, lipids, amino acids, and nucleic acids and contributes to cell signaling	[54,145]
*Biotin (B8)*	µg	2.5	5–35		Coenzyme functioning in bicarbonate-dependent carboxylations	[150]
*Folic acid (B9)*	µg	1–18	80–600		Involved in nucleic acid synthesis and a cofactor in biochemical reactions critical for the synthesis, replication, and repair of nucleotides for DNA and RNA	[54,146,147,148]
*Cobalamin (B12)*	µg	0.27–0.9	0.4–2.8	Synthesized by rumen and gut microorganisms	Coenzyme in transmethylation reactions. It is essential to the health of nerve tissue, the functioning of the brain, and the production of red blood cells	[54,149,166,167,168]
Vitamin C *Ascorbic acid*	µg	2000	25,000–70,000		Antioxidant molecule	[169]

^1^ per 100 g of whole milk; ^2^ recommended daily intake as defined in (World Health Organization, 2004, [150]), for the lesser and most demanding human physiological phase, respectively.

### 3.6. Aromatic Compounds

Flavor is defined as taste plus aroma. The perception of milk’s flavor by the consumer depends on milk components directly contributing (i.e., lactose, minerals) or serving as carriers for taste and aroma (i.e., milk fat, albumin) [55,90,170]. On top of that, a main contribution to milk flavor is given by several volatile aromatic substances (Table 6), whose abundance is mainly dependent on the feeding regime adopted on the farm (i.e., pasture and forages composition). Aldehydes are the main contributors to cow’s fresh milk aroma: 10 to 40 ppb of these compounds provides a herbaceous flavor to milk, but a penetrating off-flavor may emerge when they are present at higher concentrations [112]. Another class of volatile compounds with a major role in conferring a fruity aroma to fresh milk is esters [171], even though these compounds are destroyed during thermal process and become secondary in pasteurized milk and derivatives. Finally, an important sulfur compound representing 25% of the volatile fraction in bovine milk is dimethyl sulfone, which provides pleasant hot milk, leather, and bovine sweat-like aromas [96]. Other aromatic compounds (i.e., alcohols, ketones) have a secondary importance in determining fresh milk’s aroma under normal conditions, but their role may become relevant in processed milk and dairy products. Another class of compounds potentially revesting a considerable contribution to defining milk’s aroma when cows are raised in pastural systems are those originating from plants’ secondary metabolism (i.e., terpenes) [172]. Although the aromatic profile of milk mainly depends on nutritional practices adopted on the farm, it has never been included among the milk quality aspects that are assessed by the dairy industry.

**Table 6:** Main aromatic compounds contained in commercial cow milk and relationships linking these components to flavor perception.

Compound	Origin ^1^	Affecting Factors ^1^	Milk Type ^1^	Odor Description	Reference
Aldehydes					
*Nonanal*	Autoxidation of UFAs	NUT	PM	Sweet, floral, green, grass-like	[58,112,171,173,174]
*Hexanal*	Autoxidation of UFAs	NUT	PM	Freshly cut grass, green	[173]
*Benzothiazole*	Enzymatic AA catabolism	NUT	UHTM	Burning smell, rubbery	[58,173,175]
Esters					
*Ethyl butanoate*	Biosynthesis within the mammary gland or after milking by bacterial activity	HT	RM	Fruity, sweet, banana, fragrant	[58,112,171,173,175,176]
*Ethyl hexanoate*		HT	RM	Fruity, pineapple, apple, unripe fruit	[58,112,171,173,175,176]
Alcohol					
*1-Octen-3-ol*	UFA degradation	FAP	PM	Mushroom-like	[58,112,171]
Sulfur compounds					
*Dimethyl sulfone*	Catabolism of sulfurate AAs	NUT	RM; PM	Sulfurous, hot milk, burnt	[58,171,173,175]
Ketones					
*2-Heptanone*	β-oxidation and decarboxylation of SFAs		UHTM	Blue cheese, spicy	[58,173,174]
*2-Nonanone*			UHTM	Mustard-like, spicy	[58,173]
*2-Undecanone*			UHTM	Vegetable, floral, rose-like	[58,173]
*2-Tridecanone + δ-decalactone*			UHTM	Peach-like, floral	[58,173]

^1^ AAs: amino acids; FAs: fatty acids; UFAs: unsaturated fatty acids; SFAs: saturated fatty acids; NUT: nutrition; HT: heat treatment; FAP: fatty acids profile; PM: pasteurized milk; RM: raw milk; UHTM: ultra-high temperature milk.

## 4. Novel Milk Quality Parameters

Recently, consumers and stakeholders in the milk production chain have focused on quality aspects that extend beyond the compositional traits of milk [177]. These aspects are driven by public concerns over the environmental impact and the ethical issues associated with animal farming and milk production systems (i.e., animal welfare).

### 4.1. Environmental Impact

Public concern regarding the environmental impact caused by the milk production chain has grown considerably in recent decades. Rumen fermentation patterns accompanying cow’s productive career are involved in generating several byproducts exerting a potential greenhouse (GH) effect once released into the atmosphere (i.e., methane and carbon dioxide) [178]. Other milk-production-chain-derived factors that have a potential environmental impact are animal wastes: nitric oxide released by manure has a remarkable GH potential [179], nitrogen and P contained in wastes are potential stream water pollutants [180,181], while a portion of urea excreted with urine undergo enzymatic conversion to ammonia by the fecal microbiome and is finally released into the air as fine particulate matter [182]. Several interventions can be implemented at the farm level to reduce GH gas emissions related to rumen fermentation [183,184] and decrease environmental risks related to waste management [185]. Besides these aspects directly pertaining animal physiology and waste management strategies, the ecological footprint of the milk production chain is also influenced by the agronomical and logistical aspects related to the cultivation, handling, and transportation of the feedstuffs included in cows’ diets [186]. Milk produced in extensive organic systems is perceived by consumers as having a lower environmental impact than those obtained from herds raised under intensive conditions due to the lower request for external inputs. This is not always the case [187], and the environmental impact of traditional and organic systems cannot be generalized, as it depends on several aspects (i.e., farm location, environment, bovine breeds raised, feedstuff availability, farm management ability, etc.). To account for these aspects, the gold standard for assessing the environmental impact of milk production is through performing a life cycle assessment (LCA) of the whole productive process on a specific variable (i.e., water, GH gas emissions, land consumption, etc.). Performing a standardized LCA approach at the farm level and including this among the aspects of milk quality has the potential to encourage mitigation strategies aimed at lowering the environmental impact of milk production. Unfortunately, LCA certifications considering multiple aspect at once, aimed to “objectivize” the perception of the environmental impact behind milk production, are still lacking. Furthermore, LCA analysis does not consider the unique role played by ruminant livestock as a source of highly valuable protein for the human diet. Ruminants are symbiotically linked to a microbial ecosystem habiting their foregut, which is capable of turning indigestible carbohydrates into volatile fatty acids that cows use as energy sources. Biosynthetic pathways and transamination reactions mediated by the rumen microbiome improve the nutritional value of plant proteins ingested by the cow through enriching them with AAs that are essential to the human diet [188]. The high value behind this physiological characteristic of cows cannot be captured by any LCA approach and is still dependent on the individual consumer’s perception. In this respect, raising the consumption of plant-based milk-replacer beverages (i.e., soybean or rice milk) is often motivated by consumers’ perception that using feedstuffs that are included in dairy cows’ diet as human food would decrease the ecological footprint of milk production. A possible strategy to provide an objective tool to evaluate the ecological footprint of cow milk is the nutrient-focused LCA (nLCA) [189,190], recently endorsed by the FAO [191] and relating the environmental impact to the nutritional value of the product. Thus, including both LCA and nLCA measurements among milk quality parameters would allow for an objective evaluation of the ecological footprint of milk production and the transmission of more correct information to consumers.

### 4.2. Animal Welfare

Public concern for animal welfare is another emerging issue associated with milk production, which strongly encourages the adoption of a validated method to assess it at the farm level to include it among the new milk quality parameters. Despite this need being agreed upon by farmers, consumers, and all stakeholders along the milk production chain, methods to measure animal welfare are still a topic of extensive discussion across scientific disciplines. A growing consensus has emerged on two key points: (i) animal welfare encompasses both the physical and psychological well-being of animals [192]; and (ii) a comprehensive assessment of animal welfare requires a multifaceted approach that considers the biological functioning and natural behavior of the animal [193]. While a universally accepted definition of animal welfare remains elusive, the “Five Freedoms” framework proposed by the Farm Animal Welfare Council [194] meets widespread recognition. These include freedom from hunger and thirst; from discomfort; from pain, injury, and disease; from fear and distress; and the freedom to express natural behaviors. The concept has undergone further refinement over time [195,196], including the establishment of more specific criteria for each freedom and the introduction of the “positive welfare” concept. This latter concept emphasizes the importance of providing animals with opportunities for positive experiences, transcending the mere alleviation of suffering [197]. Nowadays, available methods for livestock welfare evaluation are based on scoring multiple indicators within a farm (i.e., environmental conditions, management practices, and rearing systems) that are considered proxy of the real animal welfare condition [198,199,200,201,202]. Generally, an overall welfare score for a farm is calculated based on the score of each indicator, but there is no consensus on the relative contribution of each indicator to defining the final score. The greatest limitation hindering these methods is the lack of any validated relationship between the indicators used and the actual welfare state of the animals, as no indicator directly reflecting the livestock welfare status has been identified yet. This gap indicates the need for further research and development of reliable measures that can accurately reflect the true welfare conditions experienced by the cow before including it as a recognized milk quality standard driving the consumer’s choice.

## 5. Conclusions

Today, the paradigm of milk quality encompasses novel compositional traits and even extends beyond nutrient composition, including ethical aspects such as animal welfare, the environmental impact, and the sector’s overall sustainability. The determination of several parameters proposed in the present review as potential milk quality factors would be hindered by methodological limitations in practical conditions, as their assessment would involve many analyses (some of them through technically complicated methods), and a standardized approach to integrating all these aspects into a comprehensive milk quality assessment is yet to be established. Despite that, just recognizing these as shared quality aspects by all the stakeholders (i.e., producers, processors, and consumers) will accelerate their implementation throughout the milk production chain, with expected benefits for the dairy industry of tomorrow.

## Data Availability

The original contributions presented in the study are included in the article, further inquiries can be directed to the corresponding author.
